# How Should We Deal with Neoplastic Disease and Serious Infections Caused by Epstein–Barr Virus?

**DOI:** 10.3390/cancers15112889

**Published:** 2023-05-24

**Authors:** Hironori Yoshiyama, Asuka Nanbo, Tomoharu Yasuda

**Affiliations:** 1 Department of Microbiology, Shimane University Faculty of Medicine, Shimane 693-8501, Japan; 2 National Research Center for the Control and Prevention of Infectious Diseases, Nagasaki University, Nagasaki 852-8523, Japan; nanboa@nagasaki-u.ac.jp; 3 Department of Immunology, Graduate School of Biomedical and Health Sciences, Hiroshima University, 1-2-3 Kasumi, Minami-ku, Hiroshima 734-8551, Japan; yasudat@hiroshima-u.ac.jp

Epstein–Barr virus (EBV) is a ubiquitous herpesvirus, but also the first discovered human tumor virus [1]. EBV is transmitted by saliva exchange, and in primary infections the virus spreads rapidly in the body and sometimes presents with symptoms of infectious mononucleosis [2]. However, once acquired immunity to EBV antigens is established, EBV changes its life cycle from lytic infection to latent infection, and employs a survival strategy of amplifying the genome through division and proliferation of latently infected cells. Latent infection expresses a limited number of viral genes that are required for maintenance of the episomal genome in the host cell nucleus. Out of 85 total viral genes, about 10 genes at most, including non-coding RNAs, are expressed during latent infection [3]. Since viral latent genes activate the proliferation of infected cells, the EBV genome propagates as the infected host cells divide and multiply. Therefore, the proliferation of B lymphocytes, T lymphocytes, natural killer (NK) cells, and epithelial cells persistently infected with EBV is responsible for the development of EBV-related malignancies [4].

However, although nearly 90% of the world’s population is infected with EBV, tumors develop only in a limited number of populations, and many of the mechanisms of tumor development associated with EBV infection are still unknown [5]. We believe cancers associated with EBV infection include many effective preventive measures, which include a potential target for novel cancer diagnostics, therapeutic approaches, and the possibility of prevention by vaccination. A brief summary of all accepted papers is provided below.

Tamura Y et al. formed an international joint research team consisting of Japan and Germany. The team confirmed that helper T cells activated by recognizing EBV-infected cells convert into killer T cells that are functionally different from the original helper T cells [6]. Furthermore, the team revealed that the entire gene region of killer T cells derived from such helper T cells is subject to transcriptional regulation that is very similar to that of the original killer T cells. This finding suggests that T cells may recognize a wider range of epitopes to eliminate virus-infected cells than previously thought. Since such a phenomenon does not apply to the conventional functional classification of T cells, reconsideration of functional classification of T cells will be required in the future.

Heawchaiyaphum C et al. performed a panel of transcriptome analyses of EBV-associated epithelial carcinomas (EBVaCA), including nasopharyngeal carcinoma (NPC), EBV-associated gastric carcinoma (EBVaGC), and oral squamous cell carcinoma (OSCC), and identified a genetic signature and common features associated with EBV infection [7]. By examining changes in gene expression levels in EBV-infected cell lines and tumor tissues, Heawchaiyaphum C et al. found that two genes, SLC26A9 and TMC8, are upregulated. Furthermore, both SLC26A9 and TMC8 are differentially expressed genes in EBV-infected cells, and were highly correlated with the activation of genes involved in many biological processes and pathways, including IL6/JAK/STAT3 and TNF-α/ NF-κB signaling pathways. The study may serve as a novel and reliable tool for predicting the patients’ prognosis and establishing the treatment strategies.

EBV can infect not only B cells and epithelial cells, but T cells and NK cells. Relatively rare but fatal diseases such as nasal extranodal NK/T-cell lymphoma, malignant NK-cell leukemia, and chronic active EBV infection are also EBV-related malignant diseases. These diseases exhibit systemic inflammation and sometimes progress to hemophagocytic lymphohistiocytosis (HLH), a life-threatening immune overload condition. Preventing the development of HLH is important for the treatment of EBV-associated malignant diseases derived from NK/T cells. Yoshimori M et al. showed that suppression of IFN-γ may regulate HLH in EBV-positive NK cell tumors [8]. This new finding will lead to the development of new treatments for EBV-associated malignant diseases originated from EBV-infected NK/T-cells. 

Nasopharyngeal carcinoma is distinguished from other head and neck cancers in that EBV infection is associated with carcinogenesis. Nasopharyngeal carcinoma is highly metastatic, and development of a novel therapeutic modality for metastatic nasopharyngeal carcinoma is keenly awaited. Protein farnesylation, a C-terminal lipid modification of proteins, was initially investigated as a key process that activates RAS oncoproteins through association with cell membrane structures. Since then, more and more evidence has accumulated that proteins other than RAS are also farnesylated and play an important role in carcinogenesis. Kobayashi E et al. reviews the molecular pathogenesis of protein farnesylation in nasopharyngeal carcinoma and discusses the potential of farnesylation as a therapeutic target [9].

New insights into basic and translational knowledge about EBV-related diseases were presented at the ‘19th International Symposium on Epstein–Barr Virus and Related Diseases’, held 29–30 July 2021, in Asahikawa, Japan. The proceedings of this international conference notes that basic and translational discoveries regarding EBV-associated tumors, including NK/T-cell lymphoma, gastric cancer, and nasopharyngeal carcinoma, were presented by an international group of scientists and clinicians, among others [10].

In summary, this Special Issue of *Cancers* collects a wide range of knowledge from the pathogenesis, treatment, and prevention of EBV-related malignancies as original research papers and original review articles covering not only basic medicine but also clinical medicine.
